# Endoscopic resection of an inverted Meckel's diverticulum manifesting as a pedunculated lesion with a double-lumen sign

**DOI:** 10.1055/a-2913-8714

**Published:** 2026-08-17

**Authors:** Jing Guo, Guanqi Fan, Zongling Lv, Xiuli Zuo

**Affiliations:** 1 Department of Gastroenterology 91623 Qilu Hospital of Shandong UniversityJinanShandong ProvinceChina; 2 Department of Radiology 91623 Qilu Hospital of Shandong UniversityJinanShandong ProvinceChina; 3 Department of Pathology 91623 Qilu Hospital of Shandong UniversityJinanShandong ProvinceChina

**Fig. 1:**
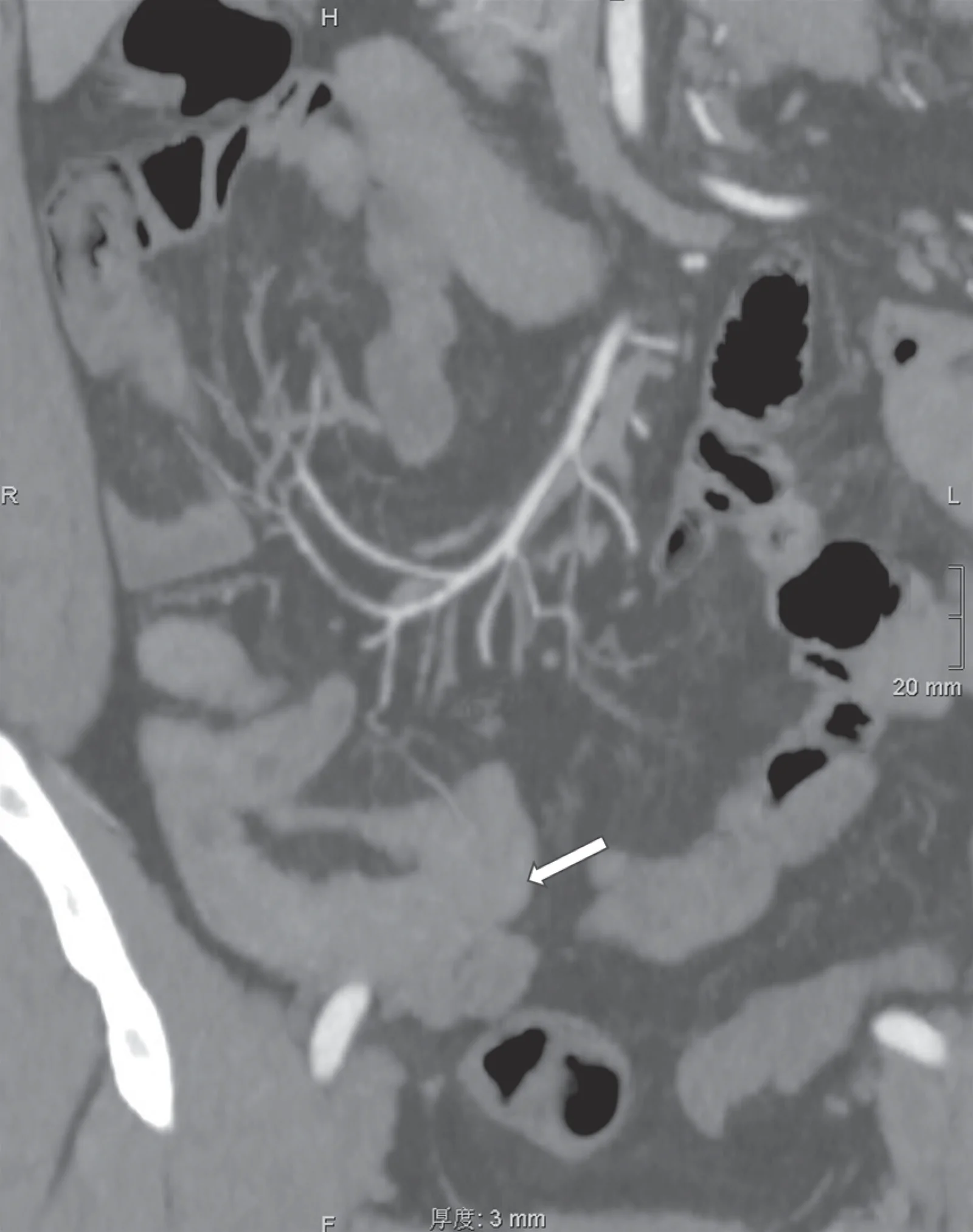
A computed tomography (CT) image showing a strip-like soft tissue shadow within the ileum in the right lower quadrant (arrow).

**Fig. 2:**
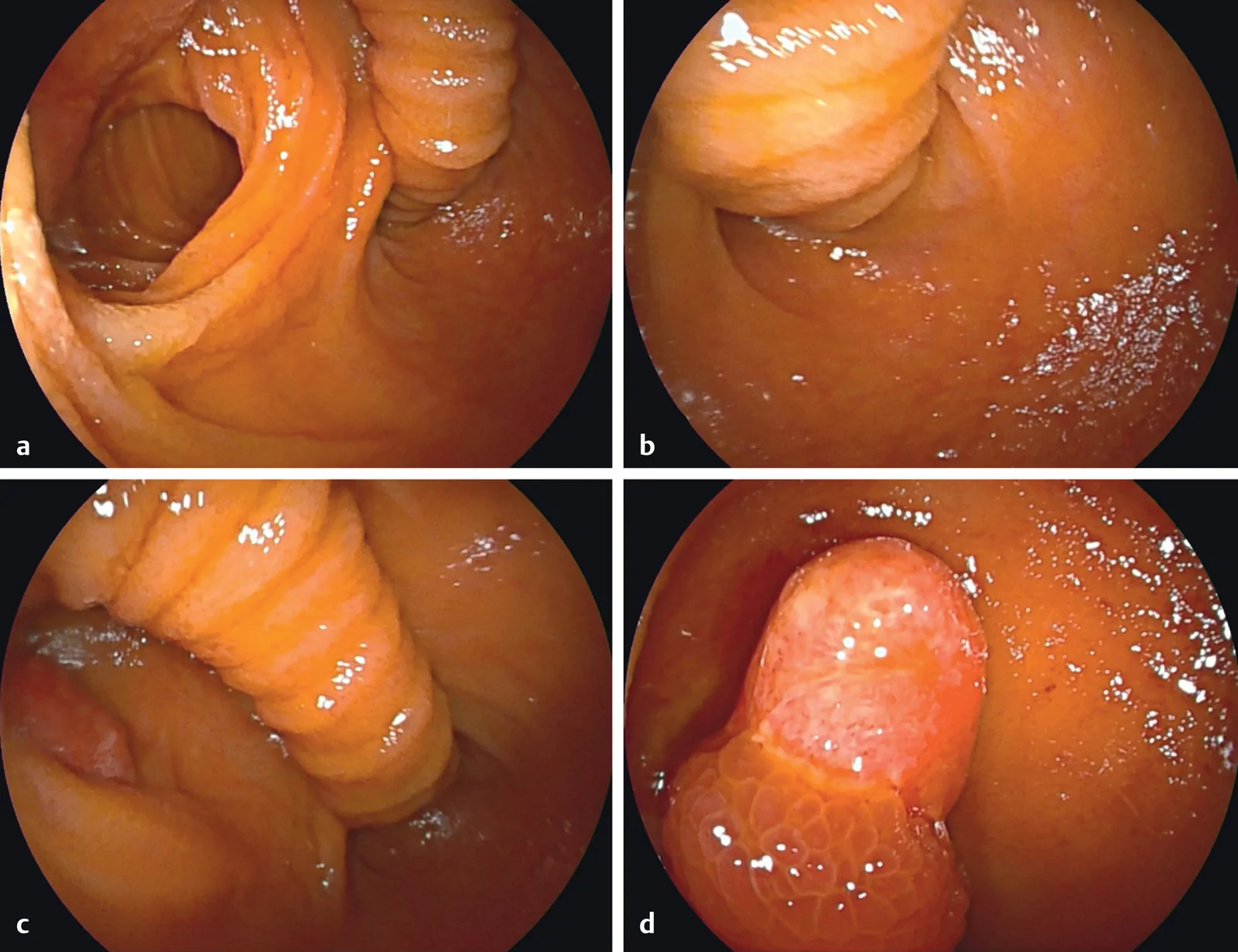
Endoscopic features of an inverted Meckel's diverticulum. ( **a** ) The “double-lumen” sign observed in the ileum. ( **b** ) A pedunculated lesion arising from one lumen, with its base continuous with the normal small intestinal mucosa. ( **c** ) The lesion exhibited a fold-like stalk. ( **d** ) The head of the lesion was rough and edematous with ulceration.

**Fig. 3:**
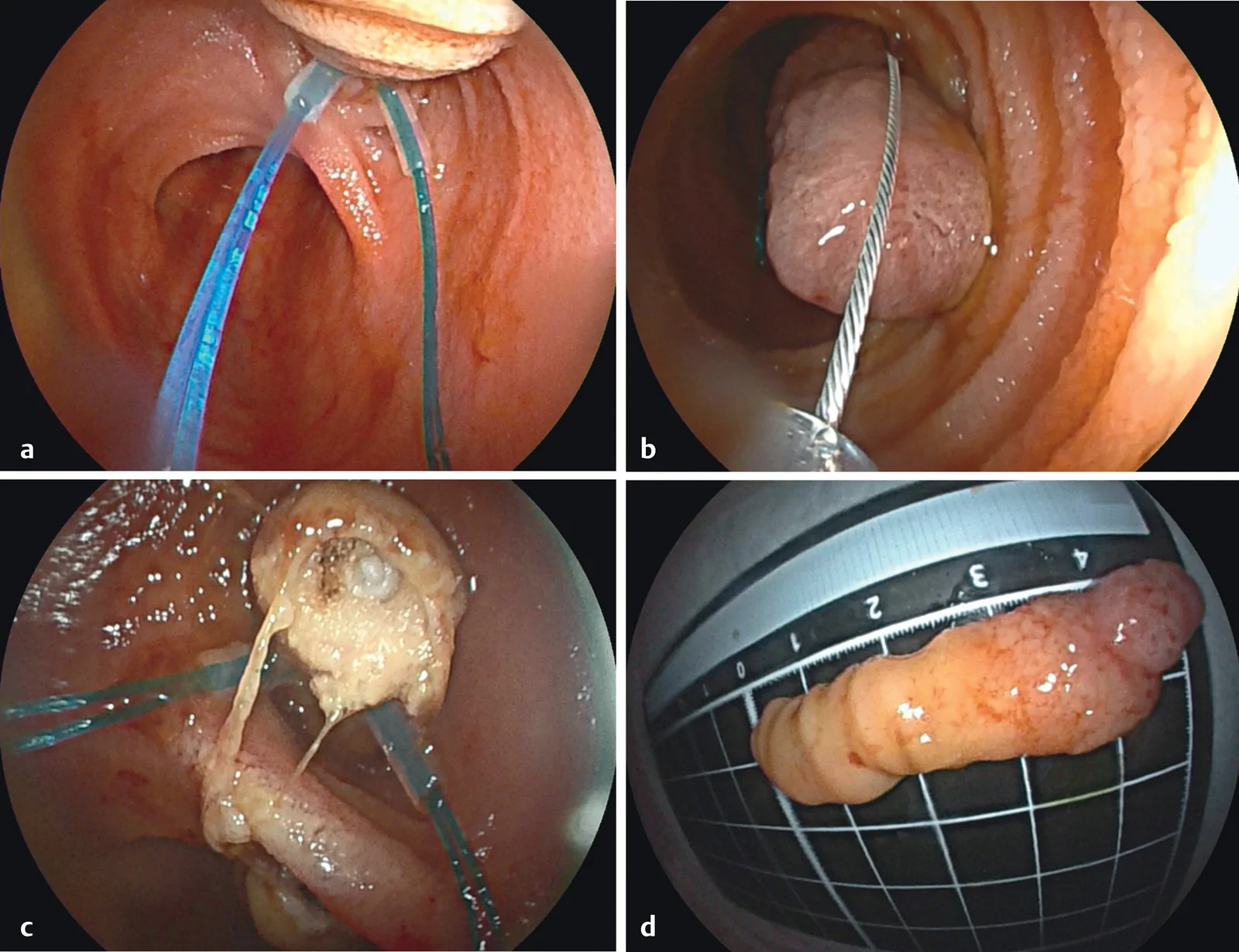
An endoscopic full-thickness resection procedure. ( **a** ) Two sequential detachable snares ligating the base of the inverted diverticulum. ( **b** ) Snare polypectomy performed after the lesion turning cyanotic. ( **c** ) The post-resection surface without prophylactic clipping. ( **d** ) The resected specimen measuring 5 cm in length.

**Fig. 4:**
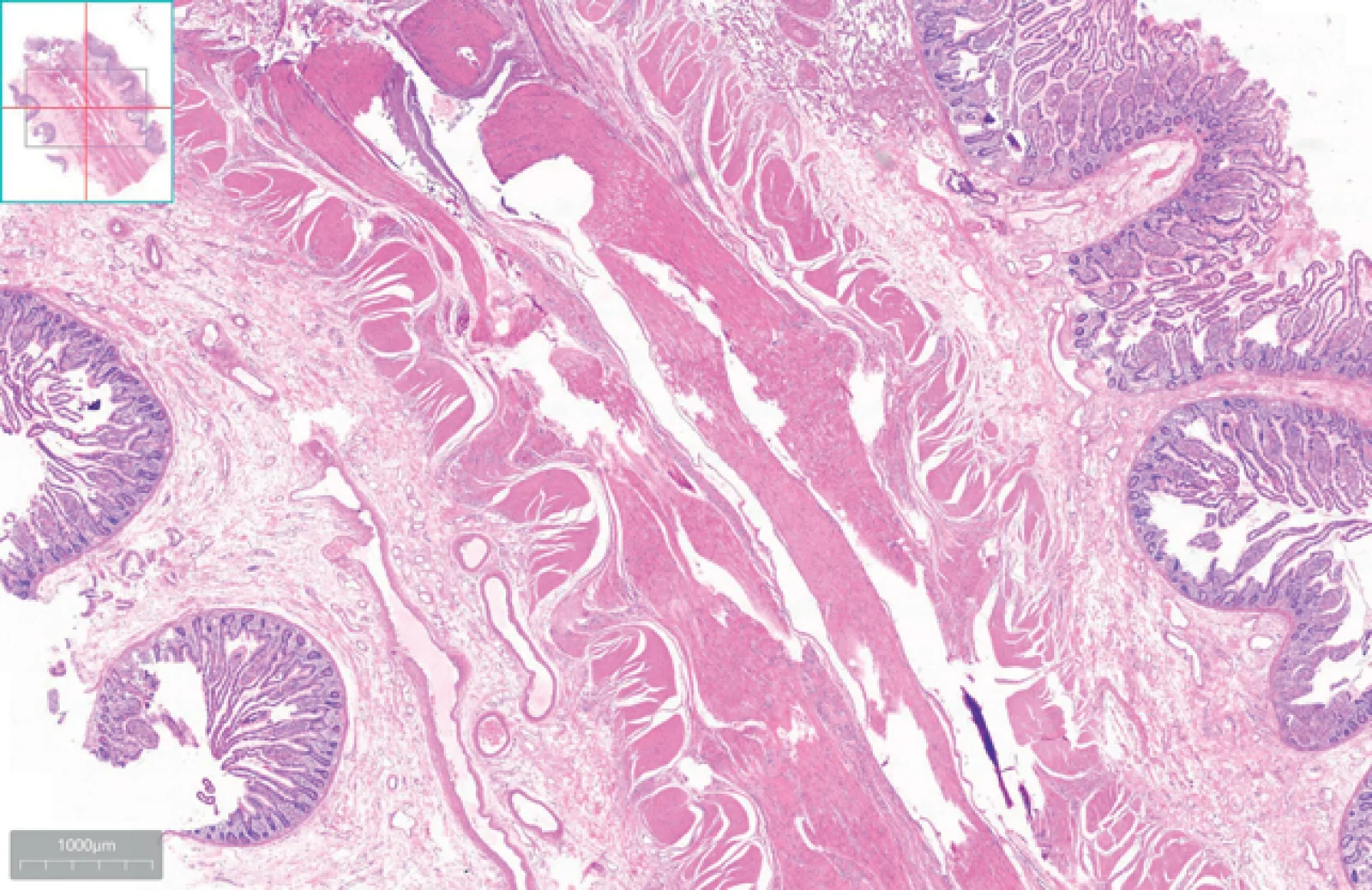
Pathological examination confirmed an inverted Meckel's diverticulum without ectopic tissue. Microscopy showed two layers of full-thickness intestinal wall, with localized mucosal ulceration.

A 57-year-old man presented with a 10-day history of melena and hematochezia. He had been on aspirin for 6 months post-stroke. Laboratory tests showed severe anemia (hemoglobin 50 g/L), and blood transfusions were administered. Esophagogastroduodenoscopy and colonoscopy showed no abnormalities. Computed tomography (CT) revealed a strip-like soft tissue shadow within the ileum ( Fig. 1 ). Double-balloon enteroscopy (DBE) was performed via the anal route. Approximately 80 cm proximal to the ileocecal valve, a “double-lumen” sign was observed ( Fig. 2a ). A pedunculated lesion arose from one lumen, featuring a base continuous with the small intestinal mucosa ( Fig. 2b ), a fold-like stalk ( Fig. 2c ), and a rough, edematous head with ulceration ( Fig. 2d ). The above features suggested an inverted Meckel's diverticulum (IMD); thus, endoscopic full-thickness resection was attempted. Two detachable snares were used sequentially to ligate the base of the IMD, and the lesion gradually turned cyanotic, suggesting effective ligation. Then, snare polypectomy was performed proximal to the detachable snares ( Fig. 3a–d , Video 1 ). Pathological examination confirmed a 5 cm IMD ( Fig. 4 ). The patient was discharged 4 days postoperatively.

**Video 1** An endoscopic view showing the “double-lumen” sign and a pedunculated lesion arising from one lumen, followed by sequential detachable snare-assisted, clip-free full-thickness resection of an inverted Meckel's diverticulum. Video URI:


The IMD is rare and difficult to diagnose, often mimicking a pedunculated polyp or submucosal tumor; intraprocedural diagnosis has relied on indirect findings such as mucosal features or cross-sectional imaging. 1​ This case presents the direct visualization of the double-lumen sign, with a prolapsed lesion arising from one lumen continuous with the normal small intestinal mucosa with a fold-like stalk and a head with ulceration, which we propose as the typical manifestation of the partial or incomplete IMD. Although DBE has facilitated endoscopic resection in selected cases, 2​ we achieved endoscopic resection using pre-ligation with two detachable snares without prophylactic clipping, and we propose this clip-free technique as safe, efficient, and economical. Together, this report presents the most characteristic diagnostic findings and the least invasive therapeutic approach for IMD to date.

UCTN_Code_TTT_1AQ_2AD_3AB
